# Quality Improvement Programme on Implementing Co-Production in Care Programme Approach in an In-Patient Rehabilitation Psychiatric Unit to Enhance Patient Engagement and Positive Step-Down Discharges

**DOI:** 10.1192/j.eurpsy.2024.1481

**Published:** 2024-08-27

**Authors:** H. Tahseen, A. Hassoulas, K. Umla-Runge

**Affiliations:** ^1^Adult Psychiatry, Cygnet Healthcare, Mold; ^2^School of Medicine, Cardiff University, Cardiff, United Kingdom

## Abstract

**Introduction:**

This quality improvement (QI) programme was proposed to integrate co-production principles into rehabilitation psychiatry, focusing on enhancing patient-centred care and promoting positive step-down discharges within the mental health system. The backdrop of the QI programme was the essential role of rehabilitation psychiatry in aiding the recovery and reintegration of individuals with mental health challenges, and sub-optimal audit results about patient’s attendance and positive step-down discharges at an in-patient psychiatric unit.

**Objectives:**

The QI programme aimed to implement and explore Co-production, a transformative approach involving patients and healthcare professionals as equal partners.

- To promote co-production in psychiatric in-patient service
- To improve patient experience in the CPA meetings
- To reduce anxiety associated with the CPA meetings and discharge planning
- To assess staff’s limitations and barriers in promoting co-production.

**Methods:**

The QI programme was divided into phases, including diagnostic, problem-solving, and evaluation. It employed diagnostic tools such as the fishbone cause and effect diagram and the 5-Why Technique for root cause analysis. The project’s aim was aligned with the Model of Improvement, guided by the three fundamental questions. Change ideas were developed using driver’s diagram and were then evaluated through PDSA cycles. Quantitative analysis utilized paired t-tests to assess the significance of changes, and qualitative analysis focused on patient perspectives gathered through the co-produced CPA questionnaire. Emerging themes from the questionnaire responses were integrated into the project’s trajectory through narrative synthesis. Predictions were formulated to measure project success: 50% patient attendance in the next CPA meetings, 70% positive step-down discharges, and improved Hamilton Anxiety Rating Scale (HAM-A) scores.

**Results:**

The iterative Plan-Do-Study-Act (PDSA) cycles demonstrated the evolving impact of interventions on patient engagement and discharge outcomes. Implementation of patient information leaflets, staff training, and a CPA agenda template led to increased attendance and positive step-down discharges. Analysis of HAM-A scores revealed a substantial decline in anxiety levels for almost all participants, suggesting the effectiveness of the interventions. Discharge outcomes were influenced by patient engagement and tailored interventions. Patient responses revealed themes such as challenges during transitions to community care, empowerment from shared decision-making, and diverse experiences in communication with healthcare professionals.

**Conclusions:**

The CPA agenda template improved patient experiences by enhancing communication and patient-centeredness.

**Disclosure of Interest:**

None Declared

